# Interactive Cardio System for Healthcare Improvement

**DOI:** 10.3390/s23031186

**Published:** 2023-01-20

**Authors:** Galya Georgieva-Tsaneva

**Affiliations:** Institute of Robotics, Bulgarian Academy of Science, 1113 Sofia, Bulgaria; galicaneva@abv.bg

**Keywords:** cardiovascular diseases, computer system, ECG, PPG, Holter records, mathematical analysis, arrhythmia, heart failure, heart rate variability

## Abstract

The paper presents an interactive cardio system that can be used to improve healthcare. The proposed system receives, processes, and analyzes cardio data using an Internet-based software platform. The system enables the acquisition of biomedical data using various means of recording cardiac signals located in remote locations around the world. The recorded discretized cardio information is transmitted to the system for processing and mathematical analysis. At the same time, the recorded cardio data can also be stored online in established databases. The article presents the algorithms for the preprocessing and mathematical analysis of cardio data (heart rate variability). The results of studies conducted on the Holter recordings of healthy individuals and individuals with cardiovascular diseases are presented. The created system can be used for the remote monitoring of patients with chronic cardiovascular diseases or patients in remote settlements (where, for example, there may be no hospitals), control and assistance in the process of treatment, and monitoring the taking of prescribed drugs to help to improve people’s quality of life. In addition, the issue of ensuring the security of cardio information and the confidentiality of the personal data of health users is considered.

## 1. Introduction

Advances in technology are leading to the ever-greater miniaturization of digital sensors, leading to an increase in the possibility of ever more accurate and easy continuous monitoring of individuals, as needed, using mobile sensing devices.

The miniaturization and the development of intelligent applications make it possible to offer qualitatively new services in healthcare in which the care of the patient’s health and comfort are placed first. The development of network technologies leads to the possibility of remote medical monitoring and diagnostics even in remote settlements (which may lack hospital services) by qualified doctors. This puts healthcare on a new, modern footing, guaranteeing every patient the best healthcare services, regardless of their location.

Monitoring the activity of the cardiovascular system is an important task on which the maintenance of the good health of the individual depends. Diseases of the cardiovascular system are very common; for example, about one million new cases worldwide are registered every year. According to statistics, 35–40% of patients diagnosed with heart failure die within a year of diagnosis; by the end of the fifth year, the mortality reaches 75%.

Heart rate testing is performed by recording cardiac signals through the use of an electrocardiographic (ECG—short-term cardio records), photoplethysmographic (PPG records), or Holter device (long-term cardio records). The main method for studying heart activity is heart rate variability (HRV), an indicator of the overall health of the human organism, which is effectively used in the diagnosis and prevention of cardiac diseases as well as several other diseases.

HRV gives the most complete picture of the influence of the autonomic nervous system [1,2] (through the sympathetic and parasympathetic nerves) on cardiac activity. Variability refers to the changes in the length of the successive time intervals between individual heartbeats. By studying the mathematical parameters of HRV [3,4,5,6], a complex assessment of the individual’s health status, the work of their cardiovascular system, and the ability of their body to adapt to the adverse effects of the environment and stress are possible. An advantage of HRV parameters is that they are determined non-invasively and can be calculated in real-time.

The autonomic nervous system (ANS) directly affects cardiovascular activity, the digestive system, respiration, pupillary response, and other organs in the human body. Heart rate variability parameters can be used to assess the results of the interaction between the sympathetic and parasympathetic nervous systems [7], to screen for cardiovascular disease, depression, stress, diabetes, hypertension, and more.

Several authors who studied HRV before 1996 indicated different values for its normal parameters that correspond to a healthy organism. This necessitates the creation of a single standard for determining the parameters of HRV and standardizing the values for norm and pathology. Today, the research methods used for the analysis of the real cardiac data of patients with cardiac diseases and healthy individuals must comply with the HRV standard adopted in March 1996 by the European Society of Cardiology and the North American Society of Electrophysiology: “Heart Rate Variability—Standards of Measurement, Physiological Interpretation, and Clinical Use” [8]. The standard was confirmed in the following years as international and retains its relevance and universality to this day. It is a basis for creating devices and software programs for recording cardiac data and evaluating HRV.

Currently, there are several methods by which heart rate variations can be determined. Mathematical methods can be grouped as follows:

- Linear methods: including time domain, frequency domain, and time-frequency domain methods.
- Nonlinear methods: including fractal methods, Hurst exponent determination, Detrended Fluctuation Analysis, Poincaré plot, and others.

The most widely used are linear methods, where traditional methods of analysis in the time and frequency domain are applied. These methods are standardized in the accepted variability standard and are considered generally valid.

### 1.1. Background

The mathematical analysis of HRV is a task to which many current studies have been devoted [9,10]. HRV analysis software systems developed in recent years offer time-domain and frequency-domain variability analysis [11,12]. However, heart rate variability is not yet sufficiently well studied and is the subject of growing scientific interest.

In [13], HRV was investigated in healthy subjects and compared to patients with abnormal cardiograms. The authors of [14] conducted a study on the HRV of patients diagnosed with sleep apnea and presented the results of the influence of this disease on variability. Investigations of HRV in the time and frequency domain and the influence of the day/night cycle were conducted by Burger et al. [15], observing healthy, heart disease, and diabetes patients.

The detection and classification of heart attacks and the statistical analysis of ECG and HRV in a Matlab environment are presented in [16]. Selvaraj [17] examined the influence of six emotional states (joy, sadness, fear, surprise, disgust, and neutral) in healthy volunteers on HRV. For this purpose, HRV was studied using statistical methods and the obtained results indicated low values of HRV during strong negative emotions. Park, in [18], while applying a spectral analysis of HRV on the ECG of children, found a change in HRV during the growth of children.

Studies have shown a decrease in HRV with increasing age [19] or with an unhealthy lifestyle [20] (for example, smoking and alcohol use). In addition, low values of variability indicators have been reported in advanced hypertension, coronary heart disease, after myocardial infarction [21], heart failure [22], ischemic heart disease [23], etc. A decrease in HRV is accepted as a risk indicator for the occurrence of adverse events not only in chronically diseased people but also in healthy individuals.

A reduction in HRV measured in the time domain is a predictor of sudden cardiac death [11,24] and brain death [25]; permanently low values are reported in diabetes [26], congestive heart failure, sleep apnea, alcohol abuse, hypertension, syncope, and problems with prenatal fetal development. According to Rich et al. [27], if the value of one of the parameters (SDNN, for example) is less than 50 ms, the probability of sudden cardiac death within one year increases 18 times compared to healthy individuals with whom this parameter is within normal limits.

SDNN and other statistical parameters are independent prognostic indicators, for example, after acute myocardial infarction (MI). Patients at risk of mortality have lower values of the SDNN parameter. Follow-up of this index shows that after an experienced MI, patients with an SDNN less than 70 ms (when examined on a 24-h Holter recording) are nearly four times more likely to die in the next three years [28,29].

Low HRV is associated with a worse prognosis in the cardiac plan and the development of diseases, such as diabetes mellitus, epilepsy, Parkinson’s disease, depression, and others. Many occupations today are associated with high stress, for example, medical workers, teachers, military personnel, and others; in these occupations, a decrease in HRV and arrhythmias can be observed. The sympathetic nervous system becomes overloaded as a result of psychosocial stress and the use of certain drugs [30].

High HRV indicates the body’s adaptability, while low HRV indicates the body’s response to the negative effects of stress and diseases [31]. Some researchers believe that people are in good health when the heart, respiratory, and central nervous systems are functioning in sync. This results in good autonomic nervous system tone and high HRV. Conducting remote monitoring of patients with at-risk cardiovascular diseases should be a priority and can lead to adequate treatment, prolonging life, and ensuring a comfortable lifestyle.

An important point in the processing of cardiac data is the sampling frequency of the registered signals. The authors of [32] found a need for several times the higher sampling rate of the electrocardiogram in patients with low HRV, while in healthy subjects, a sampling rate greater than 125 Hz gives good results.

Holter monitoring is the most effective means today for the long-term continuous monitoring and evaluation of cardiac activity (providing 4 to 12 leads). In addition to it, wristbands, a ring for one of the fingers of the hand, chest straps, smart clothing with built-in sensors, and others can be used.

Photoplethysmographic signals [12,33,34] are an alternative to electrocardiological signals and are easier to record; the PPG devices [35], which capture these signals, are more convenient for longer wear. PPG sensors are small and can be conveniently integrated into various lightweight and easily portable devices, smartphones, and smartwatches. In the past few years, with the improvement of PPG sensor manufacturing and miniaturization technology, PPG technology has entered the daily life of more and more people and become an integral part of them. Today’s high-tech sensors make it possible to measure various parameters through which the overall health of the body can be observed.

**Figure 1:**
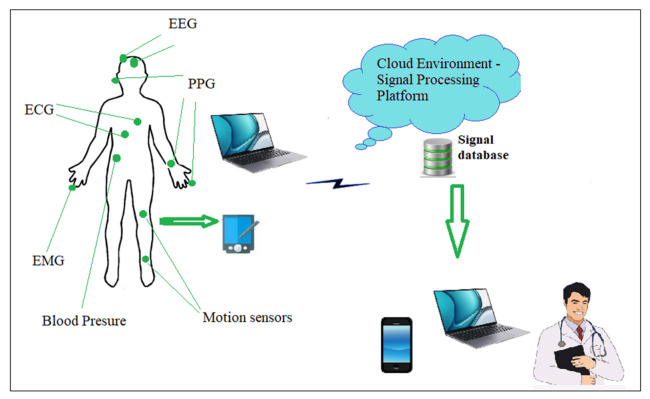
Scheme of a health system based on body sensors.

Electronic health care (e-Health) is increasingly entering the lives of ordinary people. Telemedicine systems have evolved and today are based on the Internet of Things (IoT) and are at the service of electronic and mobile healthcare. Body Sensor Networks [36] and wireless communications [35,37] are technologies providing progress in health care as well as the possibility of the effective daily monitoring of patients in various risk groups and timely medical intervention, if necessary. In Body Sensor Networks, light, wearable sensors are placed on the human body for measuring health parameters such as temperature, heart activity (ECG, PPG), breathing, and others. During a certain period, the registered signals are transmitted via the base station to a collection center server in cloud technologies which doctors can access on their devices. These systems are well suited for daily long-term remote monitoring where the individual can continue to perform their daily activities. A schematic of such a system is shown in Figure 1. Several sensors are used, placed on different places of the human body, to allow the following parameters to be registered and recorded in real-time: electrocardiogram and photoplethysmogram, oxygen in the blood, blood pressure, respiration, electroencephalogram, magnetic resonance imaging, electromyography, motion sensors. The sensors are then integrated into sensor nodes containing an analysis and control system. The registered signals are then transmitted wirelessly to a software platform based on cloud technologies, processing and analyzing the received biomedical data, which, when a risk event occurs, sends a signal to the supervising doctor. Logged data is saved to web-based data repositories.

The system includes sensors to register biomedical signals, based on which, first aid can be carried out. These signals can also be used to determine the risk that the monitored subject has developed a condition that requires emergency medical intervention and can monitor for the first signs of COVID-19 or other contagious conditions, the early detection of which can save the individual who is sick and protect others. More and more medical conditions can be treated through such networks, which will lead to fewer visits to GPs and fewer emergency and hospital admissions, leaving only those that are truly urgent, as well as an opportunity to analyze the level of urgency before arriving at the hospital. Observations on healthy people can provide information on the early signs of future diseases and the possibility of preventing health problems. The recorded signals assist the monitored subject, medical personnel arriving on the scene, and doctors who can remotely assist. The useful indicators are shown on the display along with the Global Positioning System (GPS) coordinates of the patient’s location.

The PPG recording as a parallel process of the ECG recording of heart activity can serve to increase the accuracy in locating the maximum amplitude deviations of the signal and achieve a higher reliability of the information. The parameters that are derived from the PPG are comparable to measurements that are carried out with an ECG, and scientific studies show that they correlate with traditional ECG measurements and are a good enough alternative to study cardiac activity [35].

The monitoring framework (Figure 1) includes sensor nodes where biomedical information is registered (body sensors); a communication network for the transmission of recorded signals; a server for the processing and analysis of biomedical information in remote cloud virtual centers; a database in which the information is stored; and terminal nodes (doctors, researchers) who use the information.

The following technologies can be used to analyze the collected data and make predictions: deep architectures [38,39], machine learning [40,41,42], signal processing [43], and data fusion [44,45].

In [46], a prototype of a portable system for the remote monitoring of heart rate, temperature, ECG, and electroencephalogram is presented with the parameters obtained through a wireless communication system.

Research in [47] presented a system for remote, wearable non-invasive monitoring outside of clinical settings by means of ballistocardiography (BCG) signals, with the possibility of recording the signals when the individual is standing and sitting. The research used piezoelectric film sensors to record the BCG signals and the created portable devices were tested for their level of energy consumption.

A cloud-integrated BSN (body sensor network) computing system is proposed in the work [48].

In the study [49], an automated remote cardiac monitoring system is described, including a portable ECG device, system nodes, a core processing server, data files, and a web server.

A system using cloud servers to monitor people with poor cardiovascular health was described in [50]. This system was developed using Bluetooth, an ECG sensor, Android Technology, and a web application.

### 1.2. The Purpose of This Article

In the present article, the focus is on the creation of an experimental interactive cardio system for conducting the remote monitoring of patients with risky disease states and making decisions about emergency medical intervention when necessary. The monitoring of the health status of the individual is carried out using the recording, analysis, and evaluation of cardio records (ECG, PPG, and Holter). The research conducted on cardio recordings is based on the mathematical analysis of HRV parameters by linear and non-linear methods. The studies were carried out on real Holter recordings of diseased (patients with heart failure and tachycardia) and healthy individuals. The numerical and graphical results of the conducted research are presented. The interactive system makes it possible to set the input parameters, select data for research, record the results and data obtained, and send a signal to a doctor when risky situations occur.

The goal is to study the possibilities of supporting the diagnostic process with the application of linear and non-linear methods, storing the data from the results, and giving the opportunity to monitor the development of the disease by comparing the results of successive studies.

## 2. Materials and Methods

### 2.1. Database and Preprocessing

In this study, the database used was obtained from the Medical University of Varna, Bulgaria. A total of 179 Holter recordings were studied, including recordings from patients with arrhythmia, heart failure, ischemic heart disease, syncope, and recordings from healthy individuals. The records are of men and women diagnosed by a cardiologist. The data were obtained by conducting continuous Holter monitoring (second lead). The data analyzed in the article represent long-term (approximately 24 h) data recorded from patients with a Holter device that was funded by the project [51]. The registered data were distributed according to the diagnosis made by the attending cardiologist and stored in a database [52]. The control group (volunteers) is in the same age group as the observed patients. All people participating in the study provided written informed consent to participate. Participant identification has been removed. All data used in this study are anonymized to protect the personal information of participating healthy volunteers and patients. For the present study, two groups of recordings with cardiac Holter data were selected: 12 healthy individuals, 14 patients diagnosed with heart failure, and 12 patients with tachycardia. All participants underwent continuous Holter monitoring for 24 h. This study was approved by the Research Ethics Committee at Medical University—Varna, Bulgaria, Protocol/Decision No. 82, 28 March 2019. The participants were from Varna, Bulgaria, and were aged 49 to 68 years, including both men and women. Preprocessing was performed on the recorded data, including decompression of the data (in case they were obtained in a compressed form), reduction in disturbances (removal of artifacts, filtering to reduce side noise) [53], determination of the maximum amplitude deviations in the ECG signal (R peaks) [54], extraction of RR intervals (time sequence of the time duration between adjacent R peaks), formation of the normal NN intervals, and formation of the HRV series. The resulting time series was interpolated using the cubic splines wavelet basis and downsampled at 2 Hz.

### 2.2. PPG Sensors to Heart Rate Record

To register photoplethysmographic signals at the monitoring sites of patients at risk (sensor nodes), discrete sensors such as NELLCOR DS100A [55] or compatibles can be used. To realize the recording of the analog signal, it is necessary to add a circuit for amplification, filtering, synchronization, analog-to-digital signal conversion, LED control, and microcontroller connection [56,57], such as the AFE4490 integrated circuit [58] specialized for measuring heart rate and with the possibility of measuring the content of oxygen in the blood.

The registration of the photoplethysmographic signals can be carried out and integrated into sensors. The MAX30102 integrated circuit (manufactured by the Maxim Integrated company) [59] enables short-term measurements of the heart rate and blood oxygen content. The scheme has small dimensions, low power consumption, and is inexpensive. The integrated circuit includes a red and an infrared LED to provide the necessary light for the measurements. The red LED operates at 660 nm wavelength and the infrared LED at 880 nm. The photo sensor reads the power received from the red and/or infrared LEDs. The current through them is controlled by a Pulse Width Modulator, which achieves a reduction in consumed energy.

The integrated circuit MAX30102 also includes a circuit to reduce the impact of side light, a temperature sensor for reading the temperature, analog-to-digital converters, a digital filtration module, a circuit to control the LEDs, an Interface module for connection with a microcontroller, and others.

The recorded cardio signals are processed in a portable device [60] designed for this purpose. The device checks the accuracy of the registered signals from the sensors, performs an analog-to-digital conversion of the signals, and reduces noise using filtering. The data obtained are transmitted for further processing to the created interactive software system. The information system locates the maximum deviations of the cardio signals and forms the time series of the intervals between heartbeats (HRV).

### 2.3. Mathematical Methods of Cardio Analysis

#### 2.3.1. Analysis in the Time Domain

The software system performs mathematical analysis in the time domain by calculating the static parameters HRmin, HRmax, MeanHR, MeanRR, SDNN, SDANN, RMSSD, NN50, pNN50, and SDindex (these parameters provide information on the variations between adjacent cardiac intervals). Normal values for the time indices corresponding to healthy individuals are given in the standard for HRV (Table 1).

#### 2.3.2. Analysis in the Frequency Domain

In the frequency domain, the spectral components in very low frequencies, low frequencies, and high frequencies are determined and the total spectral power is determined. Each frequency range in the long-term cardiac data has been found to have a role in the cardiac–autonomic nervous system relationship [8]:

- Ultra Low Frequency, ULF (0–0.003 Hz)—reflects the change of day and night;
- Very Low Frequency, VLF (0.003–0.04 Hz)—affects the sympathetic nervous system;
- Low Frequency, LF (0.04–0.15 Hz)—affects the sympathetic and parasympathetic nervous system;
- High Frequency, HF (0.15–0.4 Hz)—influences the parasympathetic nervous system and respiratory sinus arrhythmia;
- Total Power—reflects the influence of the two lobes of the nervous system and the overall nervous regulation of cardiac activity.

Additionally, the ratio of low-frequency components to high-frequency components (LF/HF) is determined which provides information about the sympathovagal balance of the human body. The spectral components, except in absolute units (${\mathrm{ms}}^{2}$), are also calculated in normal units (n.u).

In the frequency domain, the power spectral density (PSD) is investigated which provides information about the frequency properties of the data under investigation. PSD characterizes the intensity of the signal at different frequencies or the average power per unit bandwidth. The PSD research methods include non-parametric (Fourier transformations, Welch Periodogram, etc.) and parametric methods (autoregressive method of Burg; Periodogram method, etc.).

The PSD of an autoregressive process (Burg’s method) from p-th order is calculated by [61]:

(1) $$P_{Burg}\left(f\right)=\frac{1}{f_{s}}.\frac{\varepsilon_{p}}{{\left|1+\sum_{k=1}^{p}a_{p}\left(k\right).e^{-2\pi jkf/f_{s}}\right|}^{2}}$$

where *k*—index; *p*—the model order; $f_{s}$—sampling frequency; $\varepsilon_{p}$—least squares error; and $a_{p}$—coefficients of autoregressive Burg model.

For cardiac data (sampling rate of 2–4 Hz), the models from 16 to 20 lines are suitable [62].

Normal values for the frequency indices corresponding to healthy individuals are given in the HRV Standard (Table 2).

#### 2.3.3. Analysis in the Time-Frequency Domain

Time-frequency methods allow for visualization of the moments in time in which the frequency characteristics of the studied data change. In this study, window techniques (Window periodogram of Burg) and continuous wavelet transformation were used to determine the frequency characteristics in the time-frequency domain.

#### 2.3.4. Surface Method

The geometric method with surface determination was used, which gives us a three-dimensional view of the change in the spectral power of the signal as a function of time and frequency. The method visually represents the change in signal power, showing the exact moments in time when this happens, as well as the frequency ranges in which the spectrum changes. Wide blue areas indicate low variability of the investigated signal, which is an indicator of poor general health. Areas marked with an orange and red color signal normal (high) variability of the studied data.

#### 2.3.5. Analysis with Nonlinear Methods

In this study, the following non-linear methods [63] were used to analyze the cardiac data:

- Determination of the Hurst exponent (H) performed via the Rescaled adjusted range Statistics plot (R/S). Studies conducted on cardiac signals show that they have a fractal structure characterized by self-similarity. The degree of self-similarity can be determined by the Hurst exponent—at 0.5 < H < 1, the studied process is fractal. It was found that there is a difference in the values of this index in healthy and sick individuals. At values of H close to 1, chronic and pathological diseases are observed.
- Detrended Fluctuation Analysis (DFA). With this method, three parameters (alpha, alpha 1, and alpha 2) are determined using information obtained on the fractal correlations in the studied time series. If there is no correlation in the time series, then an alpha of less than 0.5 is obtained. At alpha > 0.5, there is a correlation dependence in the studied data. Several authors [64,65,66] have declared a difference in the values of alpha parameters in healthy and unhealthy people.

#### 2.3.6. Protection of Research Data

When transmitting and storing cardiology data, it is good to implement protection for the prevention of malicious interference and guarantee the confidentiality of the information. Cryptographic protection is applied to both the recorded cardio signal and the information part of the record containing information about the duration of the registered signal, physiological data about the patient (age, sex, weight, etc.), diagnosis, history of the disease, and others. After registering the long-term record, an encryption procedure is applied to it, including a wavelet transform on the received data sequence, optimized Energy Packing Efficiency compression, embedding a watermark in the wavelet coefficients, implementing an encryption procedure using a hybrid cryptographic algorithm, and a performing an inverse discrete wavelet transform. The technology used, and its evaluation, is described in [67].

### 2.4. Statistical Analysis

To determine the normality of the data distribution, the non-parametric Kolmogorov–Smirnov test and the Shapiro–Wilk test were used, performed using SPSS (IBM Statistics 29.0) software. The tests were applied to all the data involved in the study. The results showed that a normal distribution of the studied data can be assumed. In this study, variables and results are presented as the mean and standard deviation (mean ± sd) unless otherwise stated. From a statistical point of view, a *p*-value below which the studied parameter is considered statistically significant is important. In this study, if the *p*-value is less than or equal to 0.05 (5%), the result is considered statistically significant. Statistical analyses (between the study groups with heart disease and the healthy control group) were performed with a one-way ANOVA statistical test.

## 3. Results

The software system with which the mathematical analyses were carried out and the presented numerical and graphical results were obtained was implemented in the MATLAB development environment and was created to fulfill part of the tasks set in a research project. The system performs preprocessing of the input data, including noise reduction and detection of maximum amplitude deviations in the cardio recording (R peaks), forms the time series of RR intervals, and forms the sequence of normal cardio intervals (excluding ectopic beats, which do not originate from the sinus node). Mathematical studies and analyses are performed at these normal intervals.

The time analysis of HRV is based on the determination of statistical parameters examining changes in the duration of consecutive normal cardio intervals obtained from recorded Holter recordings. This type of analysis is performed for long recordings (approximately 24 h) through statistical and graphical measurements. The analysis in the frequency domain was performed on a 5-min part of the cardio record (according to the recommendations of the HRV standard).

### 3.1. Time Domain Methods

Table 3 presents the demographic characteristics of the examined records of the individuals, male and female, aged 42 to 68 years. The study included 14 records with a diagnosis of heart failure, 12 records with tachycardia, and 12 records of healthy volunteers. Estimates by gender and age did not show significant differences.

**Figure 2:**
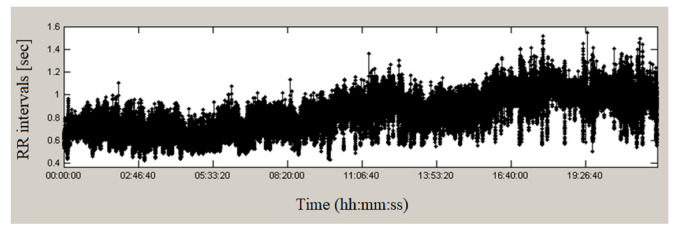
RR intervals of a healthy individual.

**Figure 3:**
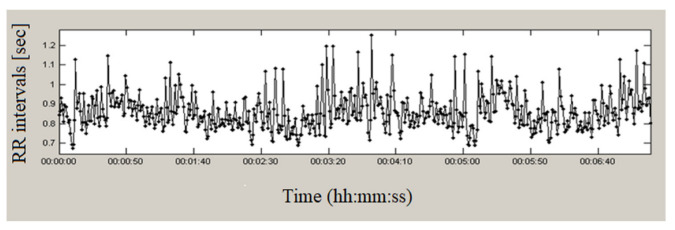
Holter record (7 min) of a healthy individual.

Figure 2 presents a continuous 24-h recording from the studied database of a healthy individual. The duration of RR intervals varies over time, having values from 0.38 s to 1.58 s, which indicates significant variations in the studied time series. Seven minutes of this series are presented in Figure 3; the graph shows a high variability of heart intervals, which is an indicator of good health.

Table 4 shows parameters in the time domain of the studied records. The results show lower values of the statistical indices SDNN, SDANN, RMSSD, NN50, and pNN50 in the diseased patients compared to the healthy control group (*p*-value < 0.05, therefore these results have statistical significance). From the geometrical parameters, HRVti shows highly reduced values in the sick patients compared to the healthy control group (*p*-value < 0.001, indicating statistical significance). Time domain studies have shown a reduction in HRV in individuals with cardiovascular disease.

**Figure 4:**
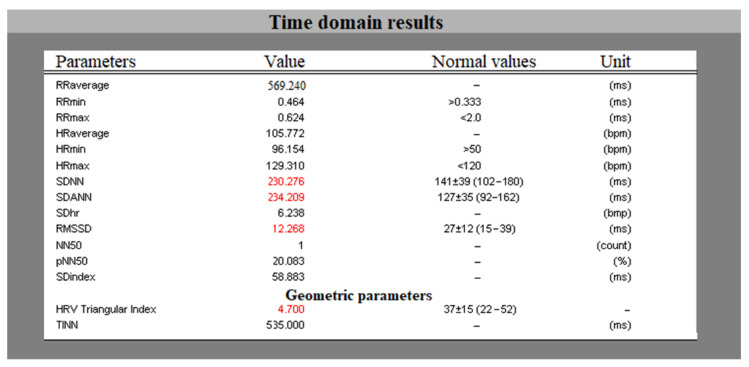
Time domain result field.

The software program created compares the results obtained in the analysis of the HRV with the reference values for the relevant parameters (according to the standard [8]) and when these values are outside the normal range for the relevant parameter, they are displayed in the table in a red color. The results of the analysis performed in the time domain as well as the reference values are displayed in the results field (Figure 4 presents the numerical results for a healthy individual on a cardiac recording obtained with a Holter device).

**Figure 5:**
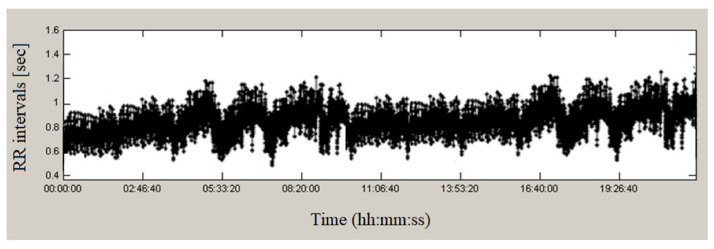
RR intervals of heart failure.

Figure 5 shows the RR time series of a Holter 24-h recording of a patient diagnosed with heart failure. The duration of RR intervals fluctuated around a mean value of 0.7 s and varied from 0.45 to 1.16 s, which, compared to the record from the control healthy group, showed a lower variability of the values of the intervals.

**Figure 6:**
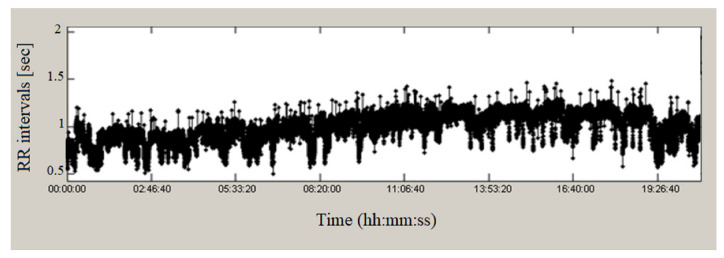
RR intervals, tachycardia.

Figure 6 presents the RR intervals of a recording of a patient with tachycardia; the duration fluctuates around a mean value of 1 s and varies from 0.5 to 1.36 s, which also shows lower variability.

**Figure 7:**
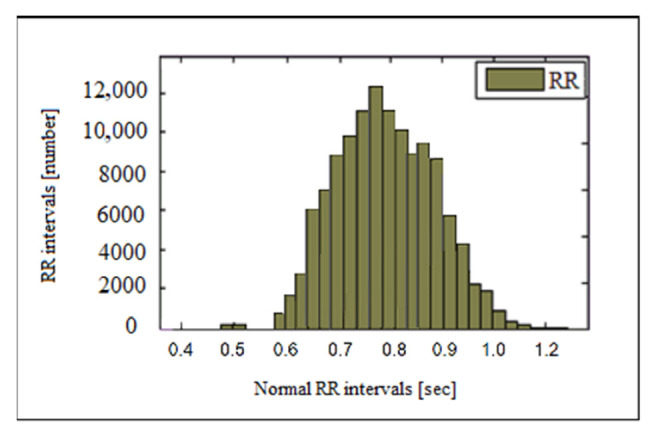
Histogram of a healthy individual.

**Figure 8:**
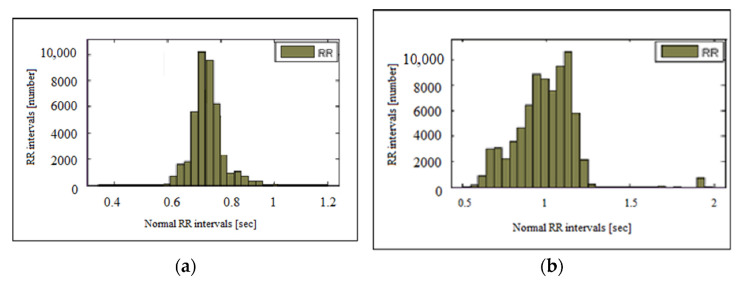
Histogram of: (**a**) heart failure and (**b**) tachycardia.

The histograms of the RR interval series and heart rate for a healthy individual and a patient with heart failure are shown in Figure 7. The histogram has the characteristic appearance of a normal Gaussian distribution, i.e., the highest bars are centrally located and relatively symmetric about the mode (0.75 s). Figure 8 presents heart failure and tachycardia histograms. The graphs show the differences in the type of histograms in healthy individuals and individuals with diseases. The histogram of an individual with heart failure has a narrow base and tall central pillars while the histogram of a patient with tachycardia is shifted to the left and has an asymmetric shape.

### 3.2. Frequency Domain Methods

In the frequency domain, the studies were performed on a five-minute series of the studied data according to the recommendations of the standard for HRV [8].

Table 5 presents the results in the frequency domain of the study conducted on recordings of healthy people and those with heart failure and tachycardia. In the healthy control group, high values of the spectral parameters can be found in the three frequency bands tested; the LF/HF ratio for the control group is 1.64, which is within the limits of the values corresponding to a good state of health according to the standard [8]. Patients with tachycardia had low properties of LF and HF spectral components and the lowest LF/HF ratio (0.96 vs. 1.64 for the healthy group, *p*-value < 0.001). These values show that tachycardia worsens the spectral HRV characteristics.

A significant decrease in LF power values was observed in patients with heart failure (482.53 vs. 1428.31 ${\mathrm{ms}}^{2}$ in the control group, *p*-value < 0.05) as well as a decrease in HF power (381.65 vs. 873.02 ${\mathrm{ms}}^{2}$ in the control group, *p*-value < 0.05). The LF and HF power values showed a 2- to 3-fold decrease in heart failure patients compared to controls.

The values of LF power, HF power, and LF/HF (ratio) have statistical significance (*p*-value < 0.05), which shows that sick individuals can be distinguished from healthy ones by these indices.

**Figure 9:**
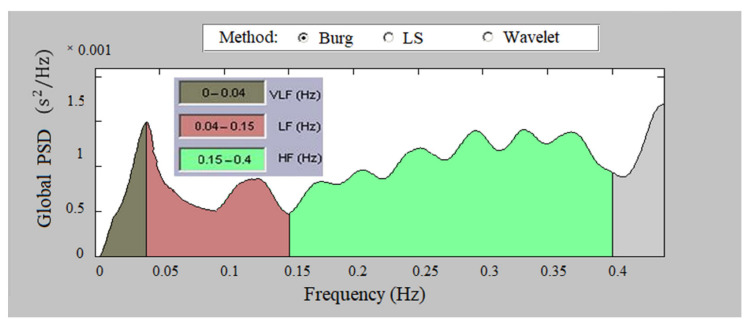
Global PSD of a healthy individual, Burg method.

The power spectral density of the signal was determined by the Burg method. The program enables its determination by the periodogram method and by the wavelet-based method. Figure 9 presents the global PSD of a healthy individual; the figure graphically illustrates high HRV values in the high-frequency and low-frequency regions in a healthy individual.

**Figure 10:**
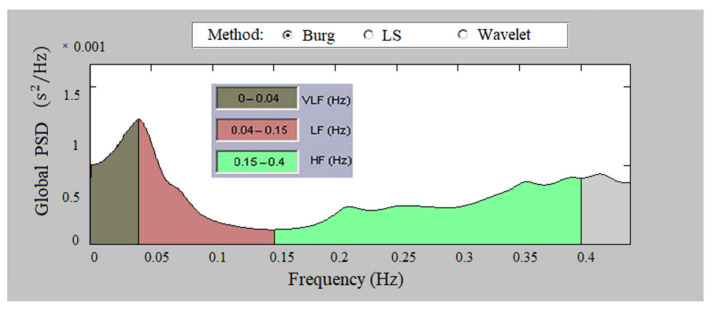
Global PSD of heart failure, Burg method.

**Figure 11:**
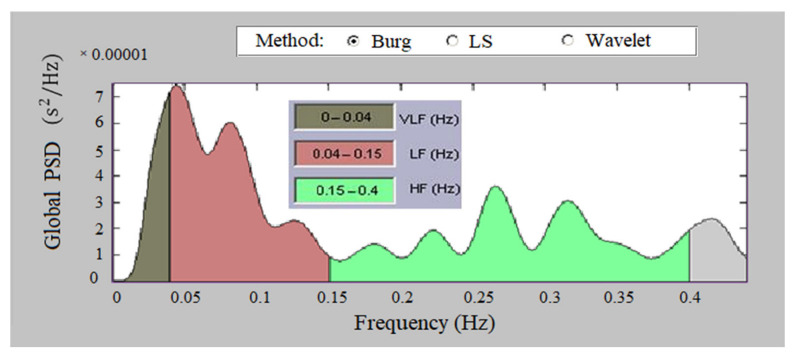
Global PSD of tachycardia, Burg method.

Figure 10 presents the global PSD of a heart failure record, according to Burg’s method. The figure shows low values of the spectral density in the high-frequency and low-frequency regions. Figure 11 shows the global PSD of tachycardia, with very low signal power values in HF and LF, which is particularly pronounced in the HF region.

**Figure 12:**
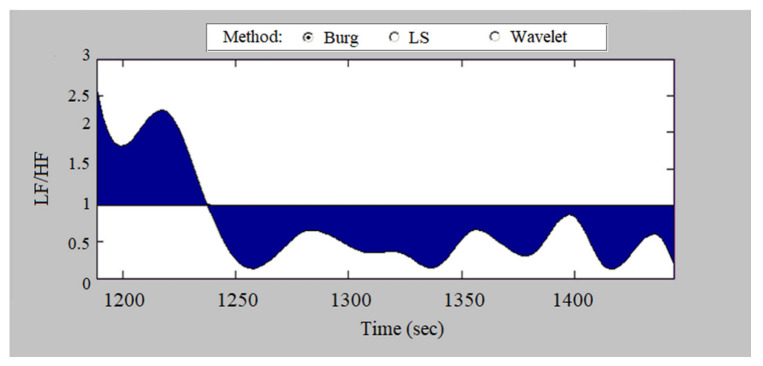
Dynamics of the values of the sympathovagal balance index (heart failure).

Figure 12 graphically presents the dynamics over time of the values of the index of sympathovagal balance in a recording of a patient with heart failure. The LF/HF values have significant fluctuations, remaining significantly lower than 1 most of the time, with an average value of 1.26 due to time intervals in which the index is significantly higher than 1. The graph shows that despite its low values this index varies over time, which is an indicator of a change in the influence of the sympathetic and parasympathetic parts of the autonomic nervous system.

### 3.3. Surface Method

**Figure 13:**
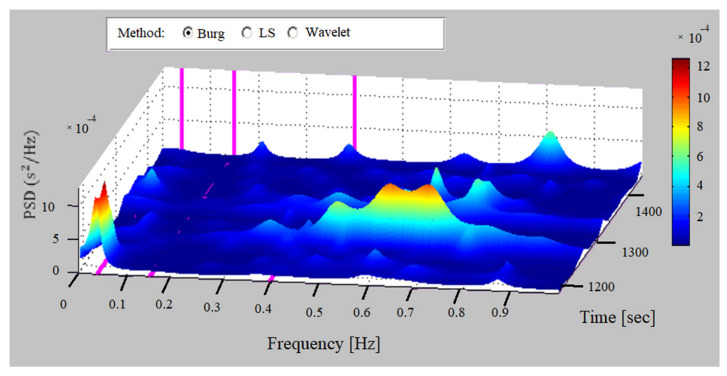
Surface method (heart failure).

The surface determination method is used to visually display the characteristics of the HRV series. Figure 13 shows a plot of the signal power spectral density surface with the Burg method of a recording from the study base of an individual with heart disease. The graph shows one peak in the frequency interval from 0.001 Hz to 0.4 Hz, which is located in the very low-frequency range. The power of the investigated signal in the remaining frequency ranges is low, with increases observed in the range of 0.5 to 0.75 Hz. The graph shows a low HRV in all studied frequency ranges throughout the studied part of the record, which is characteristic of records of individuals with cardiovascular diseases. However, in some narrow frequency ranges, distinct increases in spectral power are observed for a short time. The signal power in the low-frequency and high-frequency ranges was significantly lower than in healthy people, and the sympathovagal balance index determined by the Burg method was 1.08, well below the lower limit of 1.5 according to the standard reference values for healthy individuals (1.5–2.0).

### 3.4. Nonlinear Methods

Table 6 presents the results of the conducted research with the non-linear DFA and Hurst exponent methods. The values of alpha, alpha1, and alpha2 in healthy people differ from the values of these parameters determined in records with heart failure and tachycardia. All three DFA indices were higher in healthy individuals, the values having statistical significance (*p*-value < 0.001). The calculated value of the Hurst exponent has values higher in sick individuals (0.91 for heart failure and 0.88 for tachycardia) than in healthy individuals (0.76); Hurst exponent values close to 1 indicate the presence of health problems.

### 3.5. Examination of PPG, ECG, and Holter Signals for Health Assessment

Photoplethysmographic signals [12,33,37] are easier to register as alternatives to electrocardiological signals. The PPG devices [35,49] used to take these signals are light and comfortable for longer wear. PPG sensors are small in size and can be conveniently integrated into various lightweight and easily portable devices, smartphones, and smartwatches. Photoplethysmography determines the time between heartbeats by continuously monitoring changes in blood volume in a portion of the peripheral microvasculature. This non-invasive method of measuring pulse waves can also be the basis for the analysis of HRV. Determining one’s health status is an issue that more and more people are interested in and the study of HRV on ECG and PPG signals is the subject of diverse research interests. The miniaturization of digital sensors has led to an increase in the possibility of increasingly accurate and easy continuous monitoring of individuals as needed using mobile devices.

To account for differences between the HRV parameters, the determined PPG, ECG Holter signals, and root mean square error were used:

(2) $$\mathrm{MSE}=\sqrt{\sum_{i=1}^{N}{\left(x_{i}-y_{i}\right)}^{2\;}}$$

where i—interval index; x—first signal; y—second signal; and N—number of spaces.

**Figure 14:**
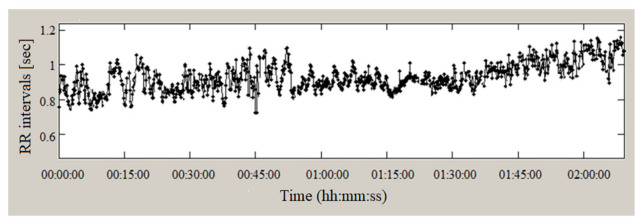
PP intervals (PPG signal, heart failure).

The cardiac records of 24 individuals (10 men and 14 women) diagnosed with heart failure and 12 records of volunteers without cardiovascular disease were examined. A Holter recording of the PP intervals (where P are the points with maximum amplitude deviations in the recording) of a PPG signal (with a duration of 2 h) is presented in Figure 14.

The results shown in Table 7 indicate a higher relative proportion of frequencies in the LF range of signal power as an indicator of patients diagnosed with heart failure when compared to the values of healthy patients. The low-frequency range reflects the influence of the sympathetic division of the nervous system. The increase in energy in the LF range is reflected in the LF/HF ratio, which respectively increases. The obtained results show that the lower activity of the sympathetic nervous system reduces the load on the heart and contributes to the normalization of its activity. With the high activity of the sympathetic nervous system, the load on the heart is high and its activity becomes difficult.

Table 8 shows the mean squared error calculated by Formula (1). Group 1—ECG data is indicated in the table with G1; G2—group 2, Holter data; G3—group 3, PPG data. The table shows the results obtained when comparing the ECG-Holter data (${\mathrm{MSE}}_{G1-G2}$), ECG-PPG (${\mathrm{MSE}}_{G1-G3}$), and Holter-PPG (${\mathrm{MSE}}_{G2-G3}$). The smaller the mean squared error (the MSE parameter), the closer the corresponding values of the two studied cardiac series are, meaning that the two studied methods for determining the temporal sequence of cardiac intervals provide similar results.

From the comparative analysis of the studied data pairs, it follows that the relative errors for all studied parameters are below 4.33% for the ECG-Holter pair, less than 6.71% for the ECG-PPG pair, and below 5.93% for the Holter-PPG pair. The calculated relative errors are small, and it can be assumed that the results obtained in the study of the HRV with the three types of signals are similar and reliable.

## 4. Discussion

Investigating the phenomenon of HRV to study various disease states is an attractive idea. Researchers are continuing to work in this direction which will lead to the expansion of the penetration of this method, which is non-invasive, inexpensive, easy to use, and suitable for clinical practice.

The research and analysis performed on three types of signals, the ECG, PPG, and Holter, show that the HRVs obtained by their use are similar, with the average value of the difference between them being 2.36%. This shows that the three types of signals give identical results and can be used equally to conduct analyses.

Holter devices are still widely used to monitor patients with at-risk cardiovascular disease. However, they are bulky and require sticking the electrodes to the human skin, which is inconvenient for prolonged use. The time is not far when they will be replaced by portable, lighter, and more flexible devices with more capabilities and conveniences connected to the Internet of Things. Studies have shown the interchangeability of ECG, PPG, and Holter records. Because of the real-time, unpredictable conditions of remote monitoring, it is good to register two or more alternative cardiac records (ECG, PPG) to ensure continuous monitoring in case of the temporary or permanent failure of any of the cardiac signal recording devices.

The presented system makes it possible to carry out mathematical studies and analyses on cardio data by means of traditional, standardized linear methods. The non-linear methods used for HRV analysis provide an additional opportunity to examine the dynamic characteristics of cardio records. Based on the analyses carried out, predictions can be made about the development of a disease and the treatment process of the disease can be supported. The created database makes it possible to store research data for one patient and monitor the development of the disease and the effect of the prescribed treatment; Furthermore, additional mathematical methods for HRV research can be added to the system. However, the operation of the software system in real-time has yet to be realized.

The obtained results from the conducted research are a contribution to the research on the phenomenon of heart rate variability and are part of the efforts to support the diagnosis, monitoring, and treatment processes of cardiac diseases (and, in particular, heart failure and tachycardia).

### 4.1. Limitations

The research conducted has certain limitations related to the small number of Holter recordings that were examined (26 recordings of individuals diagnosed with heart failure or tachycardia and 12 recordings of a healthy control group). Work is underway to expand the real database from which the Holter records are taken.

### 4.2. Future Directions

Further research will be carried out using mathematical methods based on wavelet and fractal analysis. Other graphical methods for studying cardiac activity will also be applied. The research will be performed using a photoplethysmographic method for cardio signal registration [56]. Records of individuals with various diseases (atrial fibrillation, ischemic heart disease, rhythm and conduction disorders, etc.) will also be examined. It is planned to use 3D technologies [68] as an application of virtual reality to induce stressful situations, which, in combination with analysis methods, will provide researchers with new opportunities to analyze the heart rate variability in various physiological, pathological, and stressful conditions.

## 5. Conclusions

Recent advances in technology have led to the possibility of creating body sensors to record health-important biomedical data oriented for use in high-tech environments.

The application of the body sensor network in the Internet of Things enables the continuous monitoring of patients with high-risk diseases while the patients continue to perform their daily activities. Monitoring important indicators for health makes it possible to control cardiovascular diseases as the analysis of the recorded data allows the identification of critical situations and the sending of a signal to the supervising physician.

The analysis of the set of indicators of HRV and the assessment of their dynamics in the patient’s cardiac records allows for supporting the diagnostic process and clarifying the clinical diagnosis. Today, the practical application of cardiac signal analysis methods in clinical medicine helps to solve many diagnostic-prognostic questions. The presented cardio system can improve the assessment of the health status of patients and the accuracy of the diagnosis and assist the treating cardiologist in the selection of appropriate drugs, thus shortening the decision-making time of the doctor, speeding up the healing process, and reducing the cost of treating patients.

Establishing the effective protection of data from unauthorized access through software program protection means ensuring data confidentiality. All this leads to an increase in the quality of health services.

A software system for processing and analyzing cardiac data is an opportunity to reduce the subjective factor in making a diagnosis and brings greater accuracy and objectivity to the assigned treatment. The system can also be used for preventive actions since the HRV indices change even before the risky cardiac event; this can help with the early detection of diseases and their effective treatment.

## Figures and Tables

**Table 1:** Reference values for healthy individuals in the time domain [8].

Parameters	Healthy (Mean ± SD)
Statistical parameters	
HRmin {bpm}	>50
HRmax {bpm}	<120
MeanHR {bpm}	>50, <120
MeanRR {ms}	-
SDNN {ms}	141 ± 39 (102–180)
SDANN {ms}	127 ± 35 (92–162)
RMSSD {ms}	27 ± 12 (15–39)
NN50	-
pNN50 {%}	-
SDindex {ms}	-
Geometrical parameters	
HRVti {numb}	37 ± 15 (22–52)
TINN {ms}	-

**Table 2:** Reference values for healthy individuals in the frequency domain [8].

Parameters	Frequency Range {Hz}	Healthy (Mean ± SD)
TP {ms^2^}	≤0.4	3466 ± 1018
VLF {ms^2^}	≤0.04	-
LF {ms^2^}	0.04–0.15	1170 ± 416
HF {ms^2^}	0.15–0.4	975 ± 203
LFnu {n.e.}	-	54 ± 4
HFnu {n.e.}	-	29 ± 3
LF/HF {-}	-	1.5–2.0

**Table 3:** Demographic characteristics of the examined individuals.

Parameter	Heart Failure *n* = 14	Tachycardia *n* = 12	Healthy *n* = 12	*p*-Value
Men {%}	57.14	58.33	42.67	NS
Age {years ± sd}	62.43 ± 23.08	52.28 ± 13.26	51.62 ± 20.36	NS

NS (no significance)—the value is not significant.

**Table 4:** Parameters in the time domain of the studied records.

Parameters	Heart Failure (Mean ± SD)	Tachycardia (Mean ± SD)	Healthy (Mean ± SD)	*p* Value (Mean ± SD)
Statistical parameters				
HRmin {bpm}	51 ± 13	61 ± 29	56 ± 14	NS
Hrmax {bpm}	112 ± 27	140 ± 38	103 ± 16	<0.05
MeanHR {bpm}	94.79 ± 22	103 ± 26	72 ± 26	NS
MeanRR {ms}	633.64 ± 123.86	580.56 ± 231.95	849.35 ± 321.32	NS
SDNN {ms}	82.44 ± 19.04	101.34 ± 23.63	141.82 ± 22.08	<0.001
SDANN {ms}	61.73 ± 12.92	91 ± 13.43	130.64 ± 1.5	<0.001
RMSSD {ms}	18.52 ± 2.86	8.37 ± 4.03	26.85 ± 2.3	<0.0001
NN50	640.3 ± 20.41	862.11 ± 6.06	1347.04 ± 87.36	<0.001
pNN50 {%}	14.21 ± 2.65	23.43 ± 8.15	34.92 ± 46.1	<0.001
Sdindex {ms}	56.42 ± 16.32	52.32 ± 12.03	63.04 ± 23.06	NS
Geometrical parameters				
HRVti {numb}	11.53 ± 4.02	28.43 ± 7.32	42.61 ± 14.2	<0.001
TINN {ms}	481.62 ± 61.73	420.42 ± 21.31	498.22 ± 48.09	NS

NS (no significance)—the value is not significant.

**Table 5:** Parameters in the frequency domain of the studied records.

Parameters	Heart Failure (Mean ± SD)	Tachycardia (Mean ± SD)	Healthy (Mean ± SD)	*p*-Value (Mean ± SD)
Statistical Parameters				
Total Power $\{{\mathrm{ms}}^{2}$}	12,803.92 ± 969.65	11,870.26 ± 863.14	13,921.02 ± 691.08	NS
Power VLF $\{{\mathrm{ms}}^{2}$}	11,939.57 ± 489.73	10,453.88 ± 23.75	11,620.22 ± 348.41	NS
Power LF $\{{\mathrm{ms}}^{2}$}	482.53 ± 113.06	693.71 ± 103.82	1428.31 ± 241.84	<0.05
Power HF $\{{\mathrm{ms}}^{2}$}	381.65 ± 98.55	724.85 ± 111.62	873.02 ± 183.32	<0.05
Power LF {nu}	55.85 ± 7.98	48.92 ± 10.54	62.42 ± 6.24	NS
Power HF {nu}	44.37 ± 8.71	51.11 ± 11.43	37.53 ± 4.06	NS
LF/HF (ratio)	1.26 ± 0.27	0.96 ± 0.16	1.64 ± 0.02	<0.001

NS (no significance)—the value is not significant.

**Table 6:** Parameters of the nonlinear analysis of the studied records.

Parameters	Heart Failure (Mean ± SD)	Tachycardia (Mean ± SD)	Healthy (Mean ± SD)	*p*-Value (Mean ± SD)
Statistical Parameters				
Alpha (DFA)	0.91 ± 0.36	0.83 ± 0.34	1.05 ± 0.74	<0.001
Alpha1 (DFA)	0.94 ± 0.12	0.89 ± 0.72	1.21 ± 0.83	<0.001
Alpha2 (DFA)	0.82 ± 0.03	0.64 ± 0.71	0.99 ± 0.31	<0.001
Hurst (R/S method)	0.91 ± 0.18	0.88 ± 0.11	0.76 ± 0.04	<0.001

**Table 7:** HRV parameters for ECG, Holter, and PPG records.

Parameters		Group 1 ECG (Mean ± SD)	Group 2 Holter (Mean ± SD)	Group 3 PPG (Mean ± SD)
Time domain	Mean RR (PP) {ms}	684.22 ± 214.68	661.33 ± 189.13	692.11 ± 223.83
	SDNN {ms}	84.08 ± 16.88	82.77 ± 24.32	88.66 ± 32.09
	SDANN {ms}	72.56 ± 34.21	76.01 ± 35.43	74.67 ± 31.08
	RMSSD {ms}	13.18 ± 8.65	12.35 ± 14.15	11.06 ±18.98
	SDindex {ms}	61.33 ± 26.11	64.07 ± 22.18	63.88 ± 26.44
Frequency domain	Power VLF {ms^2^}	3098.51 ± 654.22	3127.06 ± 487.34	2995.78 ± 586.39
	Power LF {ms^2^}	688.22 ± 183.06	691.89 ± 243.99	704.05 ± 433.01
	Power HF {ms^2^}	586.23 ± 204.55	582.99 ± 244.13	602.33 ± 212.03
	Power LF {nu}	0.54 ± 0.19	0.54 ± 0.16	0.53 ± 0.87
	Power HF {nu}	0.46 ± 0.23	0.46 ± 0.43	0.47 ± 0.68
	LF/HF {-}	1.17 ± 0.78	1.19 ± 0.81	1.17 ± 0.93

**Table 8:** The relative error between the ECG, Holter, and PPG records.

Parameters		$MSE_{G1-G2}\;\{\%\}$	$MSE_{G1-G3}\;\{\%\}$	$MSE_{G2-G3}\;\{\%\}$
Time domain	MeanRR(PP) {ms}	1.34	3.31	0.6
	SDNN {ms}	0.64	1.47	0.88
	SDANN {ms}	1.49	0.69	0.83
	RMSSD {ms}	2.27	3.13	5.27
	SDindex {ms}	4.01	3.18	3.58
Frequency domain	Power VLF {ms^2^}	2.96	4.92	5.93
	Power LF {ms^2^}	3.04	4.07	1.69
	Power HF {ms^2^}	4.33	6.71	2.78
	Power LF {nu}	0.04	1.65	1.97
	Power HF {н.e}	0.1	1.37	2.06
	LF/HF {-}	0.49	0.08	1.02

## Data Availability

The cardio data that were processed for the research purposes of this paper were obtained from the Medical University of Varna, Bulgaria (available at http://hrvdata.vtlab.eu/, (accessed on 30 November 2022)).
